# Factors influencing the number of applications submitted per applicant to orthopedic residency programs

**DOI:** 10.3402/meo.v21.31865

**Published:** 2016-07-21

**Authors:** Elissa S. Finkler, Harold A. Fogel, Ellen Kroin, Stephanie Kliethermes, Karen Wu, Lukas M. Nystrom, Adam P. Schiff

**Affiliations:** 1 Department of Orthopaedic Surgery and Rehabilitation, Stritch School of Medicine, Loyola University Chicago, Chicago, IL, USA; 2 Department of Public Health Sciences, Stritch School of Medicine, Loyola University Chicago, Chicago, IL, USA

**Keywords:** orthopedic surgery, residency application, National Residency Matching Program, United States Medical Licensing Exam

## Abstract

**Background:**

From 2002 to 2014, the orthopedic surgery residency applicant pool increased by 25% while the number of applications submitted per applicant rose by 69%, resulting in an increase of 109% in the number of applications received per program.

**Objective:**

This study aimed to identify applicant factors associated with an increased number of applications to orthopedic surgery residency programs.

**Design:**

An anonymous survey was sent to all applicants applying to the orthopedic surgery residency program at Loyola University. Questions were designed to define the number of applications submitted per respondent as well as the strength of their application. Of 733 surveys sent, 140 (19.1%) responses were received.

**Setting:**

An academic institution in Maywood, IL.

**Participants:**

Fourth-year medical students applying to the orthopedic surgery residency program at Loyola University.

**Results:**

An applicant's perception of how competitive he or she was (applicants who rated themselves as ‘average’ submitted more applications than those who rated themselves as either ‘good’ or ‘outstanding’, *p*=0.001) and the number of away rotations (those who completed >2 away rotations submitted more applications, *p*=0.03) were significantly associated with an increased number of applications submitted. No other responses were found to be associated with an increased number of applications submitted.

**Conclusion:**

Less qualified candidates are not applying to significantly more programs than their more qualified counterparts. The increasing number of applications represents a financial strain on the applicant, given the costs required to apply to more programs, and a time burden on individual programs to screen increasing numbers of applicants. In order to stabilize or reverse this alarming trend, orthopedic surgery residency programs should openly disclose admission criteria to prospective candidates, and medical schools should provide additional guidance for candidates in this process.

Orthopedic surgery remains one of the most competitive residencies to match for students participating in the National Residency Matching Program (NRMP) (1–4). Over the past 12 years, the number of US and Canadian applicants to US orthopedic residency programs increased by 25% (from 901 to 1,140) while at the same time, the number of applications submitted per applicant rose by 69% (40.6–70.2) (5, 6). As a result of these increases, the average number of applications received by orthopedic residency programs increased by 109% from 248.9 to 519.5 (5, 6). This dramatic increase in the number of applications submitted per applicant and the number of applications received by orthopedic residency programs has increased the financial burden and time management concerns on the applicants and programs alike. Although there is currently no information on the financial and time burden of an increasing volume of applications, the time requirements and ability to appropriately and efficiently screen an enlarging pool of orthopedic surgery applicants have been a concern at Loyola University.

Factors that determine a successful match in orthopedic surgery have been well studied and published by NRMP (7). For example, successful match applicants in the 2014 match were offered an average of 12.1 interviews and had average United States Medical Licensing Exam (USMLE) Step 1 and Step 2 scores of 245 and 251, respectively. Confirmatory data for these metrics are the fact that probability of matching is 90% when 12 interviews are obtained and 80% when USMLE Step 1 score is 240.

The highly competitive nature of the orthopedic surgery application process and the perceived need to interview at 12 programs or more to have a >90% chance to successfully match have presumably contributed to the increasing number of applications submitted. In addition, other studies have suggested that the implementation of the 80-hour work restriction has led to an increase in applications (8, 9). The NRMP and others have previously reported on factors such as class rank, USMLE 1 scores, and the number of publications that correlate with a successful match. However, recently the effect of applicant publication volume on orthopedic residency match has been called into question. Campbell et al. concluded that ‘the average matched applicant to an orthopedic residency program publishes in the peer-reviewed literature less frequently than previously reported’ (10). In addition, factors that influence applicants’ rankings of orthopedic residency programs have been studied. In the study by Huntington et al., the most important factors affecting rank lists were perceived happiness/quality of life of current residents, resident camaraderie, and impression after an away rotation (11). However, to our knowledge, there have been no investigations to determine the rationale for the number of applications submitted to orthopedic residency programs. We hypothesized that lesser qualified applicants apply to more residency programs. Current investigation is designed to delineate which factors are associated with the number of applications submitted per applicant.

## Materials and methods

This study was reviewed by our institutional review board and classified as exempt.

### Study participants and survey administration

Three orthopedic surgery attending physicians, one statistician, and three orthopedic surgery resident physicians developed a 22-question survey (Table 1). Although not independently validated, the survey obtained basic demographic information as well as data regarding specific factors that have been correlated with a successful match according to the NRMP data; Alpha Omega Alpha (AOA) status, USMLE scores, number of publications, honors status in medicine/surgery clerkships, and the number of research/work experiences. Furthermore, candidates were asked to rate their ‘application competitiveness’ based on their own assessment as poor, average, good, or outstanding. We intentionally did not define these categories for the applicants but did evaluate their objective assessment of their ‘competitiveness’ by cross referencing factors known to be associated with highly competitive applications. Qualitative data were grouped into major categories by two of the others (EF and SK), and consensus was obtained to pool data into five categories including ‘other’ for statistical analysis to ensure adequate sample size in each category. Questions 21 and 22 were grouped together due to the low number of PhD applicants (*n*=2). The survey was sent via SurveyMonkey (SurveyMonkey, Palo Alto, CA) to all applicants of our institution's 2015 orthopedic surgery residency program with a cover letter that explained the purpose of the study, emphasizing the voluntary nature and anonymity of the study. The survey was sent thrice over an 8-week period to the applicant pool in an attempt to maximize response rate.

**Table 1:** Survey questions

1. To how many orthopedic surgery residency programs did you apply? (Note: If you applied to residencies with 5- and 6-year programs, count those as 2). Please enter a numerical value only, for example, 20.
2. What is the primary reason you did not apply to more programs?
3. What is the primary reason you did not apply to fewer programs?
4. How would you rate your competitiveness as an applicant? Outstanding/Good/Average/Poor
5. What is your age? Please enter a numerical value only.
6. What is your gender? Male/Female
7. Did you couples match? Yes/No
8. Are you an AOA? Yes/No/My school does not have an AOA
9. Did you receive honors in your surgery rotation? Yes/No/No honors at my school
10. Did you receive honors in your medicine rotation? Yes/No/No honors at my school
11. Are you from a medical school with an orthopedic residency program? Yes/No
12. What region are you from?
Northeast=Maine, New Hampshire, Vermont, Massachusetts, Connecticut, Rhode Island
Mid-Atlantic=New York, Pennsylvania, New Jersey, Delaware, Maryland, Virginia, West Virginia
Southeast=Arkansas, Louisiana, Tennessee, Mississippi, Alabama, Georgia, Florida, South Carolina, North Carolina
Midwest=Kansas, Nebraska, North Dakota, South Dakota, Minnesota, Iowa, Missouri, Wisconsin, Illinois, Indiana, Michigan, Ohio, Kentucky
Southwest=Texas, Oklahoma, Colorado, Utah, Arizona, New Mexico
West=California, Nevada
Northwest=Washington, Oregon, Idaho, Montana, Wyoming
Non-contiguous 48=Alaska and Hawaii
International=Outside of the United States
13. How many away rotations does your school allow? Please answer numerically.
14. How many ‘away’ orthopedic rotations did you complete (i.e., outside of your home rotation)? Please enter a numerical value only.
15. How many ‘away’ orthopedic rotations did you complete outside your region? Please enter a numerical value only.
16. What is your USMLE Step 1 score? Please enter a numerical value only.
17. What is your USMLE Step 2 CK score? Please enter a numerical value only.
18. How many research experiences do you have? Please enter a numerical value only.
19. How many abstracts, publications, presentations? Please enter a numerical value only.
20. Number of work experiences. Please enter a numerical value only.
21. Do you have a graduate degree in addition to your medical degree? If yes, please list:
22. Do you have a PhD?

AOA, Alpha Omega Alpha; CK, clinical knowledge; USMLE, United States Medical Licensing Exam.

### Data collection and statistical analysis

Completed surveys were compiled into a single database, and all analyses were conducted using SAS v9.4 (SAS Institute Cary, NC). Continuous variables were made into categorical variables based on cut points of interest or quantiles to ensure adequate sample sizes in each category. One-way ANOVA and Independent *t*-tests were used to compare average number of residency applications for each variable collected. In cases of low cell counts or non-normality, non-parametric analyses (Kruskal–Wallis tests and Wilcoxon Rank Sum tests) were used. Post-hoc tests, adjusted for multiple comparisons, were conducted on significant associations to determine specific associations between levels of categorical variables. Significance was assessed at *α*=0.05 level.

## Results

Of 733 anonymous electronic surveys sent, 140 responses were obtained for a 19% response rate (Table 1). Of the 140 responders, 116 (82.9%) were male and 24 (17.1%) were female. Ages ranged from 24 to 33 years. The compiled results are shown in Table 2. This entire group of 140 submitted an average of 80 applications per applicant to orthopedic residency programs.

**Table 2:** Statistical analysis of survey responses

	Number of residency programs applied to		
		
	*n* (%)	Mean (SD)	*p*
Gender (*n*=140)			
Male	116 (82.9)	79.5 (28.5)	0.94^a^
Female	24 (17.1)	80.0 (23.3)	
Age (*n*=140)			
< 27	75 (53.6)	76.6 (22.0)	0.33^b^
27–28	44 (31.4)	84.7 (31.5)	
> 28	21 (15.0)	79.6 (35.5)	
Region of origin (*n*=140)			
Northeast/Southeast/Mid-Atlantic	52 (37.1)	83.0 (32.0)	0.46^c^
Midwest	61 (43.6)	80.6 (21.4)	
Southwest/West/Northwest	19 (13.6)	70.6 (18.7)	
Non-contiguous 48/International	8 (5.7)	62.9 (51.6)	
Reason not applying to more programs (*n*=132)			
Cost	52 (39.4)	70.1 (22.5)*	0.004^c^
Interest	15 (11.4)	71.7 (16.6)**	
Location	23 (17.4)	81.1 (24.2)	
Successful match	30 (22.7)	76.2 (23.1)	
Other	12 (9.1)	111.8 (44.7)*,**	
Reason for not applying to fewer programs (*n*=138)			
Competitive residency	21 (15.2)	86.6 (29.5)	0.83^c^
Cost versus benefit	5 (3.6)	67.8 (34.1)	
Fear of not enough interviews/not matching	76 (55.1)	78.8 (25.4)	
Recommended number	8 (5.8)	72.0 (9.7)	
Other	28 (20.3)	82.7 (32.2)	
Couples match (*n*=139)			
Yes	13 (9.4)	82.9 (31.3)	0.97^d^
No	126 (90.6)	79.1 (27.7)	
AOA (*n*=140)			
Yes	43 (30.7)	75.0 (23.5)	0.12^c^
No	82 (58.6)	80.6 (29.8)	
No AOA at school	15 (10.7)	82.6 (30.3)	
Applicant competitiveness			
Poor	8 (5.7)	81.3 (27.3)	0.001^c^
Average	32 (22.7)	94.6 (26.7)*	
Good	70 (49.6)	73.0 (22.4)*	
Outstanding	31 (22.0)	76.4 (35.0)*	
Other graduate degree			
Yes	27 (19.1)	81.7 (25.1)	0.59^a^
No	114 (80.9)	78.5 (28.6)	
Medical school with OR program (*n*=140)			
Yes	126 (90.0)	79.4 (26.7)	0.61^d^
No	14 (10.0)	80.3 (37.5)	
Honors in medicine rotation			
Yes	70 (49.6)	76.9 (27.9)	0.36^c^
No	56 (39.7)	81.3 (27.8)	
No honors at school	15 (10.6)	81.3 (29.6)	
Honors in surgery rotation			
Yes	72 (51.1)	79.8 (28.4)	0.93^c^
No	54 (38.3)	77.9 (27.2)	
No honors at school	15 (10.6)	80.4 (29.8)	
Number of work experiences (*n*=140)			
0–1	29 (20.7)	78.3 (27.7)	0.90^e^
2–3	49 (35.0)	77.5 (28.6)	
4–5	38 (27.1)	79.5 (26.8)	
> 5	24 (17.1)	82.6 (30.3)	
Number of research experiences (*n*=140)			
0–1	13 (9.3)	64.5 (46.8)	0.06^c^
2–3	60 (42.9)	74.9 (23.4)	
4–5	42 (30.0)	79.6 (18.7)	
> 5	25 (17.9)	95.8 (32.5)	
Number of away OR completed			
0–1	10 (7.1)	58.4 (40.8)***	0.03^c^
2	71 (50.4)	76.4 (24.6)	
> 2	60 (42.6)	85.9 (27.4)***	
Number of away OR completed outside of region			
0	36 (25.5)	78.2 (27.7)	0.61^e^
1	43 (30.5)	76.2 (25.2)	
2	42 (29.8)	79.5 (31.5)	
> 2	20 (14.2)	86.4 (26.8)	
Number of abstracts, publications, and presentations (*n*=140)			
0–1	32 (22.9)	72.3 (28.3)	0.16^e^
2–4	45 (32.1)	77.6 (27.0)	
5–7	30 (21.4)	78.9 (20.8)	
> 7	33 (23.6)	87.7 (33.4)	
USMLE Step 1 score (*n*=140)			
< 237	35 (25.0)	84.5 (35.5)	0.24^e^
237–244	36 (25.7)	83.1 (27.2)	
245–252	37 (26.4)	74.7 (20.3)	
> 252	32 (22.9)	73.6 (26.7)	
USMLE Step 2 CK score (*n*=121)			
< 239	30 (24.8)	86.5 (40.4)	0.22^b^
240–250	31 (25.6)	79.7 (18.7)	
251–261	35 (28.9)	81.3 (29.0)	
> 262	25 (20.7)	70.4 (23.1)	

AOA, Alpha Omega Alpha; CK, clinical knowledge; SD, standard deviation; USMLE, United States Medical Licensing Exam.

a Significance (*p*) is based on Independent samples *t*-test

b One-way ANOVA with Welch correction due to unequal variances

c Kruskal–Wallis test due to non-normality or low counts

d Mann–Whitney due to non-normality or low counts

e One-way ANOVA. Post-Hoc Tests were adjusted for multiple comparisons.

* Pairwise statistically significant <0.01

** pairwise statistically significant <0.05

*** pairwise statistically significant <0.10.

Our objective analysis of the candidates’ perception of their ‘competitiveness’ revealed that higher USMLE Step 1 scores, higher USMLE Step 2 Clinical Knowledge (CK) scores, honors in medicine rotation, honors in surgery rotation, and AOA designation were significantly associated (*p*<0.0001 for each) with candidates rating their application competitiveness as ‘outstanding’ (results not shown).

Several factors were found to be significantly associated with the number of applications submitted per applicant, including candidates’ own perceptions of how competitive they were. Specifically, applicants who considered their competitiveness level as ‘average’ submitted more applications than those that ranked their application competitiveness as ‘poor’, ‘good’, or ‘outstanding’ (*p*<0.001). Candidates who ranked themselves as ‘average’ on the competitive application scale submitted an average of 94.6 (±26.7) applications per applicant compared with those who considered their application competitiveness as ‘poor’, ‘good’, or ‘outstanding’ who submitted an average of 81.3 (±27.3), 73.0 (±22.4), and 76.4 (±35.0) applications per applicant to orthopedic residency programs, respectively.

In addition, the number of away orthopedic surgery rotations completed was also associated with the number of applications submitted. Applicants who completed more than two away rotations submitted significantly more applications per applicant (85.9 (±27.4)) than those who had completed none or one rotation (58.4 applications/applicant (±40.8)) or two away rotations (76.4 applications per applicant (±24.6), *p*<0.03).

‘Reasons for not applying to more programs’ also revealed statistically significant findings in the ‘other’ category. Specifically, candidates who selected ‘other’ as a reason for not applying to more programs submitted an average of 111.8 applications per applicant, which was significantly higher than any other reason listed in the category (*p*<0.004). Specific comments listed included items such as ‘would have applied to more programs but was not eligible for military programs’.

As seen in Table 2, no other factors queried for were significantly associated with the number of applications submitted per applicant. Interestingly, ‘reasons for not applying to fewer programs’ did not reveal any statistically significant findings. However, the major theme centers around obtaining enough interviews to successfully match.

## Discussion

Findings of this study are reflective of the Electronic Residency Application Service (ERAS) data demonstrating the increasing numbers of applications per applicant to orthopedic residency programs (6). As previously noted, there are both an increasing number of applicants to orthopedic residency programs and an increasing number of applications per applicant to orthopedic surgery residency programs. The NRMP surveyed program directors of orthopedic residency programs in an effort to determine factors considered important to the programs (12, 13). In 2010, orthopedic residency programs received an average of 457 applications, invited 58 resident applicants for interviews, and ranked 45 candidates to fill four PGY-1 positions. At that time, 85% of program directors cited USMLE Step 1 target scores for interview invitation and 40% cited USMLE Step 2 (CK) target scores for interviews (12). Using a five-point ranking, many other factors (e.g., clinical rotation grades, letters of reference, and MSPE/Dean Letter) were considered important in determining interview invites. In the last program directors’ survey in 2014, 93% of program directors reported that they have target USMLE Step 1 scores when considering which applicants to interview, and 52% of program directors cited using USMLE Step 2 target scores in a similar fashion. In that survey, programs received an average of 549 applications (*N*=84), sent 72 applicants an invitation to interview, interviewed an average of 59 applicants, and ranked 51 candidates (13). Another study compared the perspectives on resident selection criteria solicited from orthopedic program directors and orthopedic residency applicants. They found that program directors valued an applicant performing a rotation at their institution, USMLE 1 score, and rank in medical school to be most important. In contrast, applicants felt the most important criteria to be a letter of recommendation from an orthopedic surgeon, USMLE 1 score, and rank in medical school (14).

Our results showed 79.5 applications submitted per applicant, consistent with 2014 ERAS data where the mean number of applications submitted per applicant was 70.1 (15). Results of this investigation refute our hypothesis that less qualified candidates (based on objective measures) apply to more programs than their more qualified counterparts. Factors usually associated with a more qualified candidate such as AOA status, honors in medicine/surgery rotation, USMLE Step 1 and Step 2 scores, number of abstracts/presentations/publications, and number of research experiences were not associated with fewer applications per applicant. We also investigated other factors that could potentially affect the number of applications submitted per applicant such as couples matching, sex, age, and region of origin, and there was no significant correlation between any of these factors and the number of applications submitted. Only completion of more than two away rotations in orthopedic surgery, an applicant's perception of his/her ‘competitiveness’, and ‘other’ reasons for not applying to more programs were associated with significantly more applications submitted per applicant. Although objective measures were associated with candidates’ perceived ‘competitiveness’ level, and perceived competitiveness was associated with the number of applications submitted, there was no significant association between these objective measures (other than number of away rotations completed) and the number of applications submitted. It is not overly surprising that candidates who completed more than two away rotations in orthopedic surgery applied to a higher number of orthopedic surgery residency programs than those who completed fewer rotations. A likely explanation would be that if candidates view themselves as less competitive, they would likely attempt to ‘audition’ at more programs in an effort to gain access to more institutions and geographies and potentially programs that would have not otherwise offered them an interview. Similarly, if applicants view themselves as less competitive than their counterparts, they will tend to apply to more programs in an attempt to find programs that may not use conventional selection ‘cut off’ criteria during the process of deciding whom to invite for an interview. This may explain why candidates who rated their application competitiveness as ‘average’ submitted statistically more applications per applicant than those who rated their application competitiveness as ‘good’ or ‘outstanding’. Although not statistically different, candidates who considered their application competitiveness as ‘average’ submitted more applications per applicant than those who rated their application competitiveness as ‘poor’. We speculate that candidates who ranked their application competitiveness as ‘poor’ applied to more than one specialty.

The issue of applicants applying to multiple specialties in the same match year (residency cross-specialty applications) was not addressed in this study. Historically, for all applicants applying to orthopedic surgery residency programs, the cross-specialty application rate has been constant at approximately 20%, and the majority of cross-specialty applications are submitted to general surgical – preliminary and categorical – programs (16).

The increasing number of applicants and applications per applicant to highly competitive residency programs is not an issue isolated solely to orthopedic surgery programs. Over the past 6 years ending in 2015, for example, dermatology has seen an increase in the average number of applications per applicant for US and Canadian graduates from 53 to 66. Over the same time period, otolaryngology saw an increase from 43 to 52 applications per applicant (17). In the preliminary data recently released by ERAS, the average number of applications per applicant for other competitive specialties such as dermatology and otolaryngology appear to have decreased in 2016 (dermatology 59.2 and otolaryngology 49.0). However, the average number of applications per applicant to orthopedic surgery continues to increase and is now at 79 (18).

Although we expected to see a decrease in the number of applications submitted by better qualified candidates, this was not the case. Even well-qualified candidates submitted large numbers of applications, presumably to ensure a threshold number of interviews that statistically provide a >90% chance of a successful match based on the NRMP data (7). This phenomenon is creating an environment of increased financial and time strain for both the applicants and programs. We believe that solutions must be sought to stabilize or reverse this trend.

Medical school deans, orthopedic program directors, and orthopedic faculty who have contact with medical students must become more transparent and take a more active role in counseling candidates who have little chance of successfully matching to pursue alternative fields. Based on the 2014 NRMP survey of residency program directors, another strategy for decreasing the number of applicants is transparency of programs that use target numbers and publishing of their cut off targets in a manner that is available to all potential candidates. This would allow applicants to understand at which programs an interview and subsequent successful match is highly unlikely.

This study has several limitations, most notably the 19% response rate. It is possible that a higher response rate may have yielded different results. Furthermore, only applicants to a single institution were surveyed, thus leaving open the potential for bias based on single institution applicants. Our program received 733 applicants of the 1,062 total *applicant pool* in academic year 2015 applying to orthopedic surgery (19). Although we had a low response rate, we believe we had sufficient penetration of the orthopedic residency applicant pool. However, future studies should consider a multi-institutional approach to the applicant pool in order to increase the response rate. In addition, the survey was anonymous which limited the ability to obtain further clarification in some areas, particularly the ‘other’ category of reasons for not applying to more programs. Since it was anonymous, we also did not have the opportunity to determine the final match status of these applicants and could not correlate an applicant's competitiveness, number of programs applied to, and the success of matching.

## Conclusion

Our investigation suggests that applicants are submitting large number of applications per applicant, irrespective of the competitiveness of the individual application. In light of the enormous financial and time burden placed on applicants and programs, we suggest that our specialty, with the support of our medical schools, seek innovative and creative ways to stabilize or reverse this trend.
